# Exploring global demographics of professionals in forensic odontology: a pilot study

**DOI:** 10.1007/s12024-025-00983-z

**Published:** 2025-05-02

**Authors:** Nikolaos Angelakopoulos, Rizky Merdietio Boedi, Nikita Polukhin, Galina Zolotenkova, Akiko Kumagai, Sudheer Babu Balla

**Affiliations:** 1 Department of Orthodontics and Dentofacial Orthopedics, University of Bern, Freiburgstrasse 7, 3010 Bern, Switzerland; 2 Department of Dentistry, Faculty of Medicine, Universitas Diponegoro, Semarang, Indonesia; 3 Department of Public Health and Medical Social Sciences, Synergy University, Moscow, Russian Federation; 4 Department of Forensic Medicine, I.M. Sechenov First Moscow State Medical University, Moscow, Russian Federation; 5 Division of Forensic Odontology and Disaster Oral Medicine, Department of Forensic Science, Iwate Medical University, Iwate, Japan; 6 La Trobe Rural Health School, La Trobe University, Bendigo, Australia

**Keywords:** Forensic dentistry, Community survey, Professional competence, Demographics

## Abstract

Forensic odontology (FO) plays a crucial role in legal and humanitarian investigations, providing expert testimony and contributing to disaster victim identification (DVI). However, comprehensive global data on the demographics, distribution, and professional activities of forensic odontologists (FOs) remain limited. This pilot study aimed to explore the global landscape of FO by examining the demographic profiles, geographic distribution, professional engagement, and career trajectories of practitioners in the field. A descriptive cross-sectional design was employed, utilizing a 26-item self-administered online questionnaire developed via Microsoft Forms. The survey, comprising both open-ended and multiple-choice questions, was disseminated globally to FOs and FO students through WhatsApp®, LinkedIn®, ResearchGate®, and professional associations' email lists. A total of 206 qualified FOs and students participated in the study. The results indicated that 40.3% of respondents practice in regions where FO is formally recognized as a specialty, with notable regional disparities. Although a significant proportion reported involvement in forensic casework, 27.7% expressed dissatisfaction with the level of support and professional recognition. One of the most pressing barriers identified was the absence of FO in undergraduate dental curricula and the limited availability of specialized training programs. The survey also underscored the diverse professional roles of FOs, including participation in court proceedings, mass disaster response, and forensic research. Key challenges reported by respondents included limited recognition of the field (21.8%), restricted career opportunities (18.4%), and inadequate access to training resources (13.1%). Additionally, specific technical challenges—such as bite mark analysis and dental age estimation—were highlighted as areas requiring further attention. This study offers valuable insights into the geographic distribution and professional scope of FOs, providing a foundation for future research with expanded outreach to ensure a more globally representative sample.

## Introduction

In the aftermath of a mass disaster, the identification of deceased victims becomes a task of paramount importance. This process is governed by the stringent protocols established by the International Criminal Police Organization (INTERPOL), which recognizes three primary identifiers: friction ridge analysis, genetic profiling, and dental records [1]. Each of these identifiers is meticulously examined on-site by specialized professionals. Among them, dental data serves as a cornerstone in the identification process, necessitating the expertise of forensic odontologists (FOs) to ensure accurate and reliable assessments [2].

Forensic odontology (FO) is a niche specialized discipline within forensic science that plays a crucial role in human identification, addressing scientific, legal, and humanitarian imperatives [3, 4]. This field is practiced by dentists who undertake advanced training and education to qualify as FOs. Given the indispensable role of FOs as key contributors to forensic investigations, pressing questions arise regarding their availability, training pathways, and the standardization of forensic odontology curricula on a global scale.

Despite the critical role of FO, comprehensive global datasets on the demographics and professional characteristics of FOs remain scarce. Essential information—such as the number of practicing FOs, their geographical distribution, scope of practice, and career trajectories—remains largely undocumented. This lack of data poses significant challenges to workforce planning, professional development, and the advancement of research within this specialized domain.

Furthermore, the absence of comprehensive global data on FO education has long impeded efforts to establish standardized training frameworks. A recent study has shed light on the existence of numerous postgraduate and specialized training programs worldwide [5]. However, despite this progress, a universally accepted curriculum or global consensus on FO training standards has yet to be established.

A comprehensive understanding of the global FO landscape is essential for identifying deficiencies in education, training, and professional practice. Ensuring that FOs receive the necessary support, standardization, and professional recognition is paramount to strengthening the field. Consequently, this research seeks to bridge these gaps by conducting a global survey, systematically gathering data on FO education, training programs, and professional recognition.

## Material and methods

### *Survey participants*

This cross-sectional descriptive research was conducted using a self-administered questionnaire created with Microsoft Forms (https://forms.office.com/Pages/). The survey comprised 26 questions, including both open- and closed-ended formats, utilizing multiple-choice and essay-style responses to gather comprehensive data on FOs demographics. Participants were instructed to answer all questions, with an estimated average completion time of 7 min. The survey was distributed globally to FOs and FO students to collect data on their practices and perspectives regarding FO. The survey link remained accessible for 30 days, expiring on June 14, 2024.

Participants included in the study were qualified FOs actively engaged in academic or clinical activities worldwide, as well as FO students. The online survey was disseminated through various communication channels, including closed professional FO WhatsApp® groups, personalized LinkedIn® messages, and emails sent to professional associations via their respective presidents, requesting them to share the survey link with their members. Individuals without a professional background as FOs or FO students would be excluded from the study. Given the nature of the survey dissemination, which allowed for potential forwarding by recipients to third parties not related to the FO field, responses would be carefully screened, and individuals with inconsistent or irrelevant answers indicating a lack of FO-related expertise would be excluded from the study.

The survey link was shared on May 14, 2024, in a closed WhatsApp® group called INFOdont, representing the International Network for Forensic Odontology. This group includes 112 members, who were encouraged to further share the survey link with their respective national communities. Personalized LinkedIn® messages were sent from the first author’s (NA) profile to 30 individuals within their professional network, encouraging participation in the survey. Additionally, 25 individualized messages were sent by the first author to researchers identified through ResearchGate® using search queries relevant keyword to search for expertise, such as “forensic odontology”, and “forensic dentistry”.

A systematic approach was taken to disseminate the survey through FO associations and scientific societies. Contact information for key individuals within these associations was obtained from the membership list on the International Organization for Forensic Odonto-Stomatology (IOFOS) website (https://iofos.eu/member-of-iofos/) (Table 1). Personalized email communications were sent to these representatives or designated contacts, requesting their assistance in sharing the survey link with their members.

**Table 1:** Associations and Societies in Forensic Odontology (updated February 2025)

Forensic odontology associations and scientific societies	
International Organization for Forensic Odonto-Stomatology—IOFOS https://iofos.eu/ ✓	Indian Association of Forensic Odontology- IAFO https://iafo.in/ ✓
American Society of Forensic Odontology- ASFO https://asfo.org/ ✓	Indo Pacific Academy of Forensic Odontology- INPAFO https://www.inpafo.in/ ✓
Australian Society of Forensic Odontology—AuSFO https://www.ausfo.org.au/ ✓	Israel National Police Volunteer Dentists Unit ✓
Austrian Society of Forensic Medicine (ÖGGM) https://oeggm.com/**?**	Italy – ProOF – Progetto Odontologia Forense (Forensic Odontology Project) https://www.proofweb.it/ ✓
Brazilian Association of Forensic Odontology, Associação Brasileira de Ética e Odontologia Legal, (ABOL) https://www.abolodontologialegal.com/ ✓	Italian Academy of Legal and Forensic Dentistry (OL-F) https://ol-f.it ✓
British Association for Forensic Odontology https://www.bafo.org.uk/ ✓	Japanese Society of Forensic Odontology Japanese Society of Forensic Dental Science (JSFDS) https://www.jsfds.com/ ✓
Croatian Association of Forensic Stomatology https://hufs.eu/ ✓	Korean Committee of Forensic Odontology/Dental Jurisprudence ✓
Danish Society for Forensic Odontology—Dansk Selskab for Retsodontologi (DSFRO) https://dsfro.dk/ ✓	The Netherlands – FMG – Forensisch Medisch Genootschap https://www.forgen.nl/ ✓
Finnish Association of Forensic Odontology ✓	New Zealand Society of Forensic Odontology https://nzsfo.org.nz/t/welcome ✓
Flemish Association of Dental Experts—Belgium https://www.tandexpert.be/ ✓	Nigerian Association of Forensic Odontology **?**
Association Francaise d’Identification Odontologique (AFIO) http://www.afioasso.org/ ✓	Norwegian Society of Forensic Odontology https://www.rettsodontologi.no/ ✓
Arbeitskreis für Forensische Odonto-Stomatologie (German Academy of Forensic Odontostomatology) https://www.akfos.com/ ✓	Forensic Odontology Society of the Philippines **?**
Icelandic Society of Forensic Odontology **?**	Polish Society of Forensic Odontology ✕
Swiss – FOCH – Forensische Zahnärzte der Schweiz (Forensic Odontologist Confoederatio Helvetica) **?**	Portuguese Association of Forensic Sciences APCF (Associação Portuguesa de Ciências Forenses) https://apcforenses.org/ ✓
Swedish Society of Forensic Odontology /www.srof.se ✓	South African Society for Forensic Odonto-Stomatology – SASFOS ✓
Thai Society of Forensic Odontology (TSFO) ✓	Sociedad de Odontoestomatólogos Forenses Iberoamericanos (SOFIA) https://www.sofia.lat/ ✓
Turkey – Society of Forensic Osteology, Odontology & Identification (ADOK) **?**	Forensic Evidence Department – United Arab Emirates **?**
Ukraine Association of Forensic Odontology (UAFO) **?**	

Active: ✓, Non-active: ✕, Unknown status: **?**

Additionally, a generic email was sent to all 340 members of the Indo-Pacific Academy of Forensic Odontology (INPAFO), whose email addresses were listed on their official website (https://www.inpafo.in/our-members). This email requested their cooperation in completing the survey. Independent of the survey, an email was sent to association representatives or designated contacts to determine the status of FO associations and societies—specifically, whether they were active or inactive. If no response was received, the status of the association was considered unknown, as indicated in Table 1.

### Ethical considerations

All participants were provided with detailed information about the research objectives and procedures before participating in the study. Informed consent was obtained electronically prior to completing the questionnaire. All data were collected anonymously and stored securely, ensuring that participants' personal information was protected. Any data that could potentially reveal their identity were handled with the utmost confidentiality. Participation in the study was entirely voluntary, and participants were free to withdraw at any stage without facing any consequences or needing to provide a reason. The outlined investigation protocol received approval from the Ethics Committee of Sechenov First Moscow State Medical University, Moscow, Russia (approval number No 10–24, dated 18/04/2024).

### Data analysis

Participants were invited to share their perspectives on the key challenges or barriers they face within the field through an open-ended question. The responses were systematically categorized into ten distinct themes, allowing for a structured quantitative analysis while preserving the integrity and depth of the original thematic content.

A descriptive statistical approach was employed to characterize the study sample, enabling a detailed analysis of the data. Frequency distributions and percentages were calculated for categorical variables, including gender, geographical distribution, professional background, and areas of expertise. For continuous variables, measures of central tendency and dispersion were calculated to summarize the data. The distribution of continuous variables was evaluated using the Shapiro–Wilk test to assess normality. Based on the results, appropriate statistical measures were applied, including the median for central tendency and the interquartile range (IQR) for variability.

Beyond descriptive analysis, inferential statistics were employed through the computation of 95% confidence intervals (95% CI [lower limit, upper limit]), enhancing the robustness of the findings and providing a more comprehensive characterization of the sample’s demographic and professional attributes.

The data analysis was conducted using IBM SPSS version 26.0. For data visualization, Python libraries, including matplotlib, pandas, geopandas, and plotly, were employed to create detailed and interactive visual representations.

## Results and discussion

### Demographics

A comprehensive survey output was obtained from 206 FOs and FO students who completed the questionnaire. The results of our online survey are presented in Table 2, which provides a detailed overview of the responses collected from participants.

**Table 2:** The distribution of respondents’ categorical variables

Variable	Category	*n*	% [95% CI]
Gender	Man	92	44.7 [38, 51.5]
	Woman	114	55.3 [48.5, 62.0]
Occupation: Are you currently practicing in forensic odontology?	Yes	161	78.2 [72.1, 83.4]
	No	45	21.8 [16.6, 27.9]
Educational Background: Was forensic odontology included in your undergraduate program training?	Yes	95	46.1 [39.4, 52.9]
	No	111	53.9 [47.1, 60.6]
Which of the following best describes your occupation/career stage?	Forensic Odontology Master Student	21	10.2 [6.6, 14.9]
	Forensic Odontology Ph.D. Student	5	2.4 [0.9, 5.2]
	Qualified Forensic Odontologist (Working Part-time/Full-time)	106	51.5 [44.7, 58.2]
	Postdoctoral researcher in Forensic Odontology	3	1.5 [0.4, 3.8]
	Academic in Forensic Odontology	37	18.0 [13.2, 23.6]
	None of the above	34	16.5 [11.9, 22.0]
Certification: Do you hold any certifications in forensic odontology?	Yes	155	75.2 [69, 80.8]
	No	51	24.8 [19.2, 31]
Years of Experience: How many years have you been practicing as a forensic odontologist?	No experience	19	9.2 [5.8, 13.7]
	Less than 1 year	7	3.4 [1.5, 6.6]
	1–5 years	26	12.6 [8.6, 17.7]
	6–10 years	32	15.5 [11.1, 20.9]
	11–15 years	38	18.4 [13.6, 24.2]
	16–25 years	45	21.8 [16.6, 27.9]
	25 + years	39	18.9 [14, 24.7]
Employment Setting: In what setting do you primarily practice forensic odontology?	Police or military agency	28	13.6 [9.4, 18.8]
	Government agency (other than police or military)	67	32.5 [26.4, 39.1]
	Academic institution	76	36.9 [30.5, 43.6]
	Independent consultant	58	28.2 [22.3, 34.6]
	Other	17	8.3 [5.1, 12.6]
	N/A	4	1.9 [0.7, 4.6]
Areas of Practice: Which areas of forensic odontology are you primarily involved in? (Check all that apply)	Bite mark analysis	88	42.7 [36.1, 49.5]
	Dental age estimation	152	73.8 [67.5, 79.4]
	Identification of human remains	176	85.4 [80.1, 89.7]
	Dental malpractice cases	60	29.1 [23.2, 35.6]
	Dental negligence claims	54	26.2 [20.6, 32.5]
	N/A	6	2.9 [1.2, 5.9]
Frequency of Casework: How often do you perform forensic odontology casework?	Rarely	36	17.5 [12.8, 23.1]
	Occasionally	56	27.2 [21.5, 33.5]
	Monthly	33	16.0 [11.5, 21.5]
	Weekly	36	17.5 [12.8, 23.1]
	Daily	35	17.0 [12.3, 22.6]
	N/A	10	4.9 [2.5, 8.4]
Involvement in Court Testimony: Have you ever been involved in court testimony as a forensic odontologist?	Yes	94	45.6 [38.9, 52.5]
	No	106	51.5 [44.7, 58.2]
	N/A	6	2.9 [1.2, 5.9]
Reason to provide Court Testimony: Reason to provide Court Testimony: What is the most common issue for which you are requested to appear before the court? Please indicate the number of cases for which you were requested to appear in court. (*n* = 94)	Bite mark analysis	24	25.5 [17.6, 35.0]
	Age estimation	20	21.3 [13.9, 30.3]
	Identification of human remains	47	50.0 [40.0, 60.0]
	Dental malpractice cases	14	14.9 [8.8, 23.1]
	Child abuse	2	2.1 [0.4, 6.6]
	Injuries	7	7.4 [3.4, 14.1]
	Liability	2	2.1 [0.4, 6.6]
	Sexual Assault	2	2.1 [0.4, 6.6]
	N/A	0	0 [0, 0]
DVI Employment During Mass Disaster: Have you ever been employed as a DVI (Disaster Victim Identification) team member during a mass disaster event in your country?	Yes	104	50.5 [43.7, 57.3]
	No	99	48.1 [41.3, 54.9]
	N/A	3	1.5 [0.4, 3.8]
DVI Employment During Mass Disaster: Have you ever been employed as a DVI (Disaster Victim Identification) team member during a mass disaster event internationally?	Yes	56	27.2 [21.5, 33.5]
	No	146	70.9 [64.4, 76.8]
	N/A	4	1.9 [0.7, 4.6]
Reading Habits: How often do you typically read scientific papers related to forensic odontology?	Never	2	1.0 [0.2, 3.1]
	Occasionally	64	31.1 [25.0, 37.6]
	Monthly	47	22.8 [17.5, 28.9]
	Weekly	70	34.0 [27.8, 40.6]
	Daily	23	11.2 [7.4, 16.0]
	N/A	0	0 [0, 0]
Research Interest: Are you currently involved in any research related to forensic odontology?	No, not currently involved in research	95	46.1 [39.4, 52.9]
	Yes, collaborating on research projects	47	22.8 [17.5, 28.9]
	Yes, actively conducting research	64	31.1 [25, 37.6]
Publication History: During your tenure as a forensic odontologist, how many publications have you authored or co-authored?	None	68	33 [26.9, 39.6]
	1–5 publications	78	37.9 [31.4, 44.6]
	6–10 publications	16	7.8 [4.7, 12.0]
	11–15 publications	9	4.4 [2.2, 7.8]
	More than 15 publications	35	17.0 [12.3, 22.6]
Membership in Professional Associations: Are you a member of any international professional associations related to forensic odontology?	Yes	155	75.6 [69.4, 81.1]
	No	50	24.4 [18.9, 30.6]
Membership in professional associations (*n* = 155)	International Organization for Forensic Odonto-Stomatology (IOFOS)	60	38.7 [31.3, 46.5]
	American Society of Forensic Odontology (ASFO)	65	41.9 [34.4, 49.8]
	Australian Society of Forensic Odontology (AuSFO)	14	9.0 [5.3, 14.3]
	British Association for Forensic Odontology (BAFO)	15	9.7 [5.8, 15.1]
	Indian Association of Forensic Odontology (IBFO)	15	9.7 [5.8, 15.1]
	Other	63	40.6 [33.1, 48.5]
	N/A	0	0 [0, 0]
Satisfaction with Support and Recognition: How satisfied are you with the support and recognition of forensic odontology within the broader dental and forensic science communities?	Very dissatisfied	17	8.3 [5.1, 12.6]
	Somewhat dissatisfied	40	19.4 [14.5, 25.2]
	Neither satisfied nor dissatisfied	38	18.4 [13.6, 24.2]
	Somewhat satisfied	75	36.4 [30.1, 43.1]
	Very satisfied	34	16.5 [11.9, 22.0]
	N/A	2	1.0 [0.2, 3.1]
Recommending Forensic Odontology as a career: Would you recommend forensic odontology as a career path to aspiring dental students or professionals?	No	32	15.5 [11.1, 20.9]
	Yes, with reservations	122	59.2 [52.4, 65.8]
	Yes, highly recommend	52	25.2 [19.7, 31.5]
Forensic Odontology: Recognized Specialty or Subdiscipline? Is Forensic Odontology recognized as a dental specialty under your country's jurisdiction	Yes	83	40.3 [33.8, 47.1]
	No	123	59.7 [52.9, 66.2]
The Foremost Challenge in Forensic Odontology: What do you consider as the most significant challenge in Forensic Odontology field?	Lack of Recognition and Awareness	45	21.8 [16.6, 27.9]
	Education and Training	27	13.1 [9.0, 18.2]
	Career and Job Opportunities	38	18.4 [13.6, 24.2]
	Bitemark Analysis	17	8.3 [5.1, 12.6]
	Dental Age Estimation	8	3.9 [1.9, 7.2]
	Resources and Funding	20	9.7 [6.2, 14.3]
	Collaboration and Standardization	22	10.7 [7.0, 15.4]
	Technology and Future of Forensic Odontology	15	7.3 [4.3, 11.4]
	Identification	6	2.9 [1.2, 5.9]
	N/A	8	3.9 [1.9, 7.2]

One hundred and sixty-one participants indicated they were currently practicing in FO (78.2%, 95% CI [72.1%, 83.4%]). Among them, the proportion of females was greater (55.3%, 95% CI [48.5%, 62.0%]) than that of males. This finding is noteworthy given the historical context of FO, where foundational figures such as Dr. Oscar Amoedo, regarded as the "father" of forensic odontology [6], and Dr. Keiser-Nielsen, who coined the term "forensic odontology" [7], were both male. However, a recent study [8] has highlighted the growing historical contributions of female practitioners in FO and explored how their roles have evolved in the field over time.

**Fig. 1:**
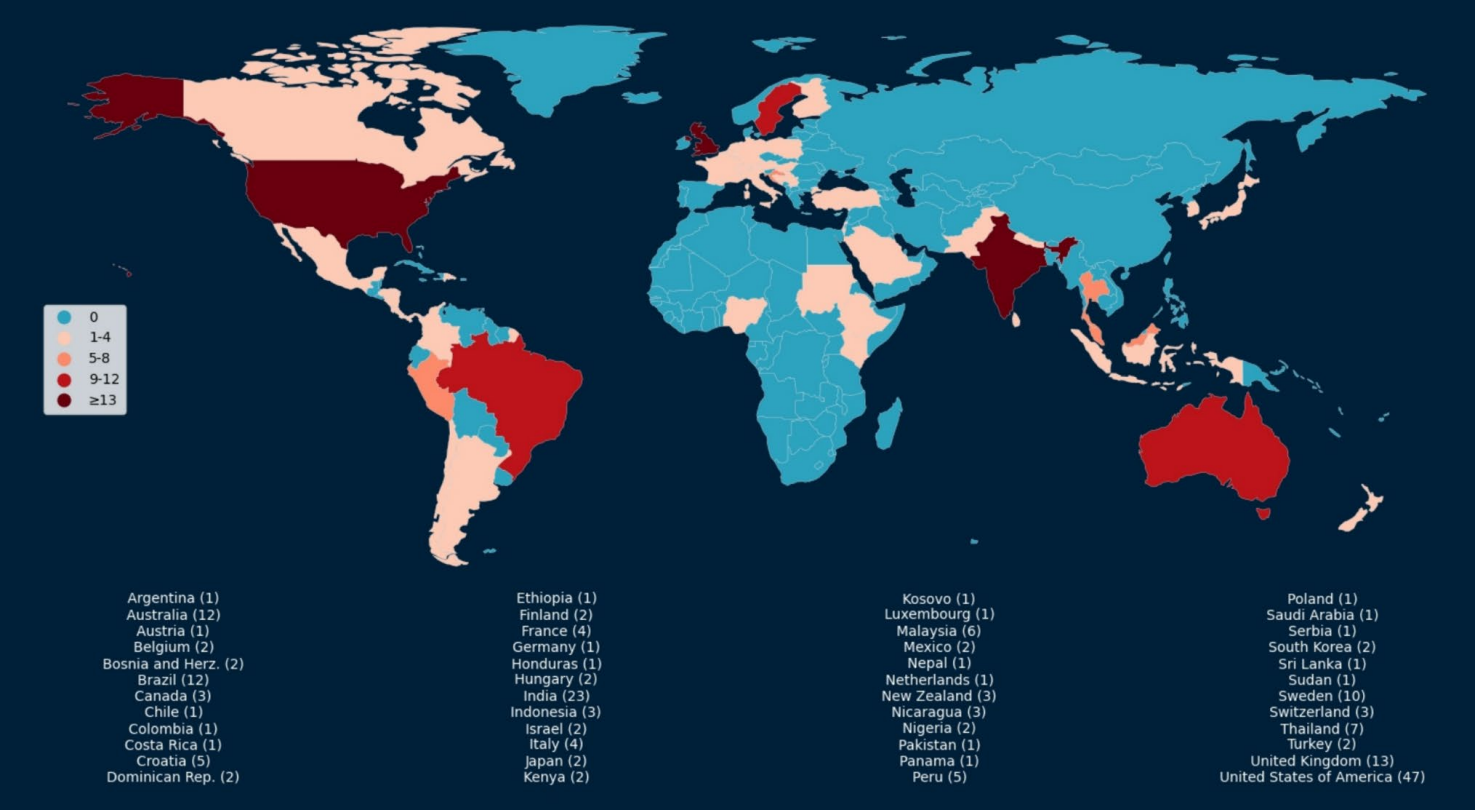
Number of survey participants by country

As shown in Fig. 1, the majority of FOs were located in the United States of America (22.8%, 95% CI [17.5%, 28.9%]), India (11.2%, 95% CI [7.4%, 16.0%]), and the United Kingdom (6.3%, 95% CI [3.6%, 10.3%]). According to a recent study [5] analyzing postgraduate and training programs in FO globally, some countries identified in their analysis were not represented in our survey responses. This discrepancy can likely be attributed to the lack of standardization in the curriculum or the fact that some countries do not offer FO training at the master’s degree level, which typically requires a longer commitment. The absence of a standardized educational path results in varied perspectives on FO practice. In some countries, practitioners are permitted to engage in FO with only basic training, while others mandate a master's degree for professional practice [5].

**Fig. 2:**
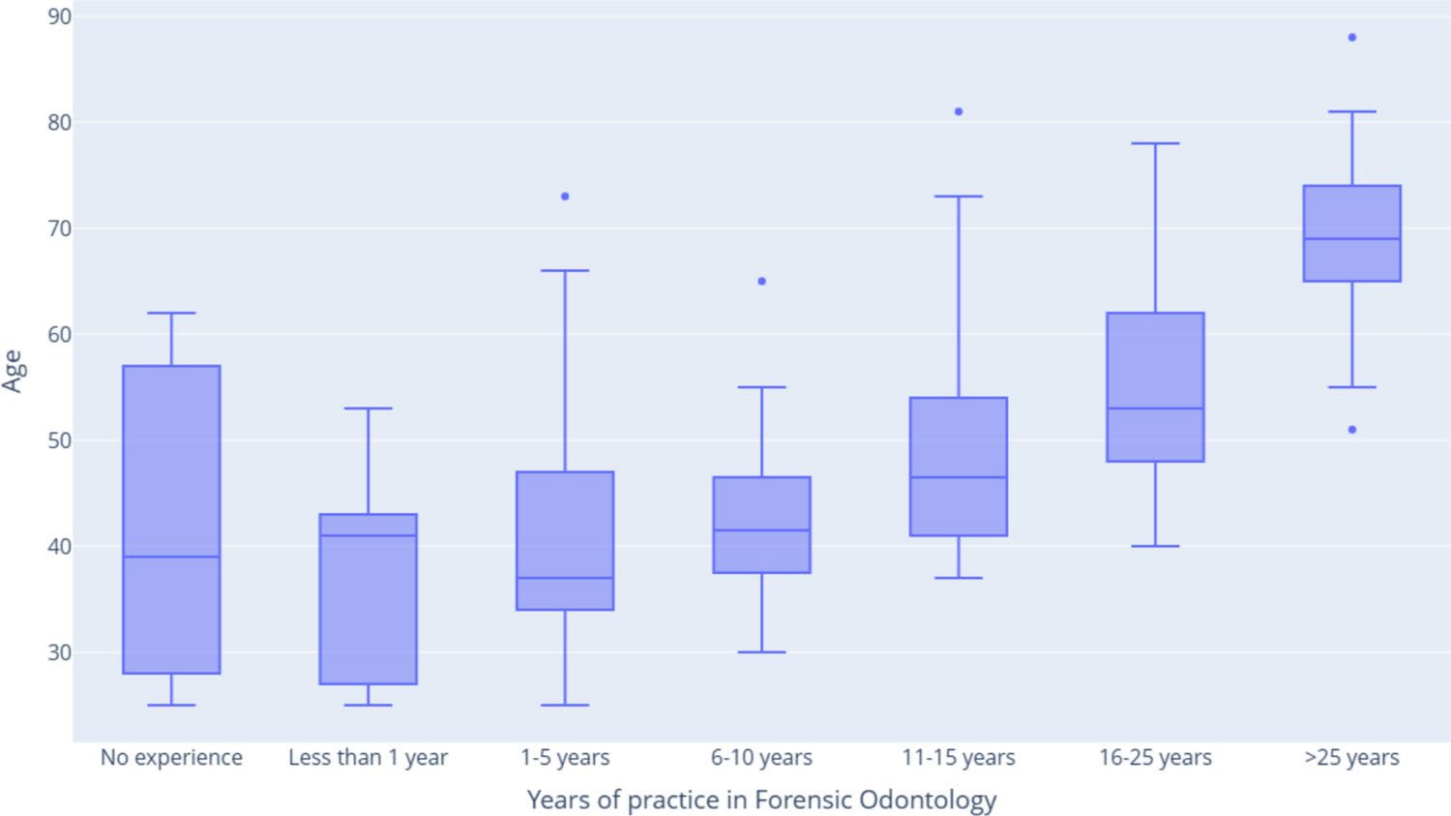
The distribution of age across the experience spectrum of FO groups

The results of the survey showed that the age of FOs was not normally distributed (W = 0.98, *p* = 0,005). It ranged from 25 to 88 years, with a median age of 49.5 years (IQR: 40–62, 95% CI [47, 53]). The age range of survey participants spans both novice and experienced professionals, which can facilitate valuable knowledge transfer and succession planning within the field of FO. However, the upper end of the age spectrum underscores the importance of targeted recruitment and the development of younger professionals. As depicted in Fig. 2, the age distribution relative to years of experience shows a gradual increase in age, from those with one to five years of practice to those with over twenty-five years of experience. Interestingly, respondents with no experience or less than one year of experience were found to have ages comparable to those of specialists with six to ten years of experience. This pattern suggests that some specialists may be entering the FO field later in their careers, possibly due to career transitions or the completion of extended academic programs.

### Education

Among the forty-five participants who reported not currently engaging in the practice of FO (21.8%, 95% CI [16.6%, 27.9%]), the majority were either enrolled in master’s programs (22.2%, 95% CI [12.0%, 35.8%]) or did not specify their occupational status (48.9%, 95% CI [34.7%, 63.2%]). Even so, a significant portion of respondents (53.0%, 95% CI [47.1%, 60.6%]) reported that FO was not included in their undergraduate training or was omitted from the dental curriculum. This observation highlights the critical need for a more comprehensive integration of FO into the curriculum, starting as early as undergraduate dental education, as emphasized by other studies [5, 9].

At the European University Cyprus (EUC) School of Dentistry, FO has been successfully integrated into the undergraduate curriculum, even within the context of a newly established dental program [10]. As part of their forensic training, dental students participate in anthropological analysis sessions, where they learn to establish biological profiles for unidentified individuals. However, the incorporation of FO into dental curricula remains limited in many developing countries, where awareness of the specialty is generally low [11–14]. Currently, no globally standardized training pathway exists for those seeking to become experts in FO, which highlights the urgent need for international efforts to standardize and expand training opportunities in this critical field.

Nonetheless, regarding actual occupation and current practice, the vast majority (75.2%, 95% CI [69.0%, 80.8%]) of the surveyed sample reported holding a certification in FO. Our findings shed light on an important aspect of the FO profession: a significant majority (75%) of FO practitioners surveyed have pursued additional postgraduate courses or specialized training to enter the field. However, this statistic also carries a dual interpretation. It suggests that 25% of current FO practitioners may have gained expertise through self-teaching or mentorship from more experienced colleagues, without having attended formal postgraduate programs. While structured postgraduate programs have existed for some time, they remain relatively limited in number, serving as the primary pathway for individuals interested in specializing in FO. A recent study that reviewed websites of FO training programs identified 56 programs across 18 countries [5]. Given the diverse paths into the field, there is an urgent need for the global standardization of both undergraduate and postgraduate training in FO. It is crucial that aspiring FOs hold a dental degree as their foundational education before pursuing specialization in this field. Standardization would not only ensure a more uniform level of expertise but also elevate the credibility and recognition of FO worldwide.

### Working experience

Respondents had a wide range of work experience, with a total of 40.7% having over 16 years of working experience. This finding suggests that the field of FO is predominantly composed of experienced professionals. However, it is important to note that 25.2% of the respondents had less than five years of experience or were new to the field. This indicates a growing influx of newer practitioners. Addressing these discrepancies is crucial as the profession progresses, particularly in terms of knowledge transfer and facilitating smooth transitions.

To achieve this, it is essential to implement mechanisms that facilitate the documentation and sharing of the expertise held by experienced professionals. These mechanisms could include mentorship programs, where seasoned professionals guide newcomers; interviews and oral histories to capture invaluable insights and experiences; written guides and best practice documents that serve as educational resources; and transitional roles, such as part-time consulting, that allow for a gradual shift from full-time responsibilities. By establishing such frameworks, the knowledge and skills of veteran practitioners can be effectively passed down, ensuring the continuity and evolution of best practices within FO.

These full-time responsibilities can largely be transferred to academic institutions, which represent the largest group of respondents’ primary workplaces (36.9%). This growing interest in academia is also reflected in the respondents' reading habits: only 1% reported never reading scientific articles, with the majority (34%) indicating that they read at least one scientific article each week. A Brazilian study [15] that examined the reading habits of FOs involved in dental age estimation found that most practitioners read at least one scientific article per month. In addition to academia, other primary employment settings included government agencies, independent consultancy roles, and police or military agencies. This diversity of employment settings highlights the multifaceted and interconnected nature of FO, encompassing areas such as education, investigations, and legal expertise, each of which plays a vital role in the practice of FO.

### Area of practice

The primary areas of practice for FOs include a number of specialties. Analysis of human remains was a predominant area, with 85.4% (95% CI [80.1%, 89.7%]) of FOs engaged in this work. Dental age estimation was also widely practiced, with 73.8% (95% CI [67.5%, 79.4%]) of FOs involved. Additionally, 42.7% (95% CI [36.1%, 49.5%]) of FOs worked with bite mark analysis. Other areas of practice included dental malpractice (29.1%, 95% CI [23.2%, 35.6%]) and dental negligence (26.2%, 95% CI [20.6%, 32.5%]). A small percentage of participants (2.9%, 95% CI [1.2%, 5.9%]) indicated that none of the above areas were applicable.

The casework frequency among respondents was mainly occasional (27.2%, 95% CI [21.5%, 33.5%]), with 17.5% rarely engaging in casework. This aligns with the finding that many FOs work in academic settings. However, FO activities are highly varied, often extending beyond the classroom to include crucial roles such as providing court testimony or being deployed in disaster victim identification (DVI) operations. These diverse functions underscore the versatility and critical nature of the profession, with FOs often bridging the gap between science, law enforcement, and humanitarian efforts.

**Fig. 3:**
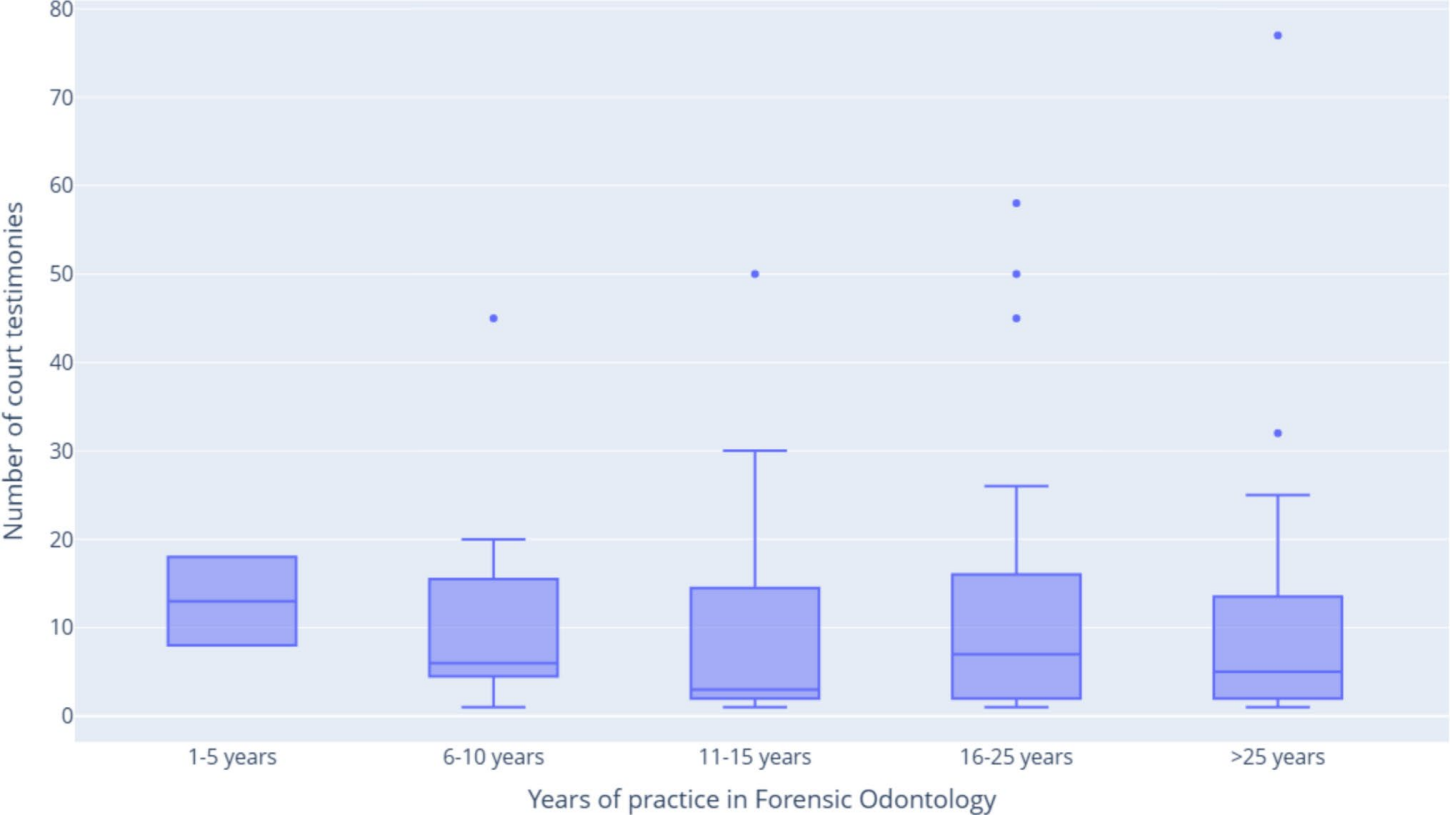
The number of court testimonies across the experience spectrum of FO groups

A notable number of respondents (45.6%, 95% CI [38.9%, 52.5%]) reported involvement in court testimony. The number of cases in which FOs were requested to appear in court was not normally distributed (W = 0.71, *p* < 0,001). The median number of cases was 6 (IQR: 2–15, 95% CI [4, 10]). As illustrated in Fig. 3, the median number of court testimonies remained consistent across experience groups. This observation may suggest that the skills necessary to testify effectively in court are typically acquired early in an FO's career. Subsequent experience likely contributes more to an FO’s ability to handle more complex or challenging cases rather than increasing the frequency of testimony.

The involvement of FOs in court testimony was driven by a range of factors. Among the FOs who provided testimony (*n* = 94), 50.0% (95% CI [40.0%, 60.0%]) of their statements were for identification purposes. Another notable proportion (25.5%, 95% CI [17.6%, 35.0%]) pertained to bite marks. Furthermore, 21.3% (95% CI [13.9%, 30.3%]) of FOs’ court testimonies pertained to dental age estimation. Other reasons included dental malpractice (14.9%, 95% CI [8.8%, 23.1%]), as well as lesser frequencies of various injuries (7.4%, 95% CI [3.4%, 14.1%]), and each of child abuse, liability, and sexual assault (2.1%, 95% CI [0.4%, 6.6%] each). These factors underscore the diverse and essential role of FOs in the legal process, particularly in cases where dental expertise is vital. Their work often carries significant legal weight, as FOs may be called upon to provide expert testimony in both civil and criminal cases [16]. The necessity of this expertise highlights the importance of well-rounded training for FOs, with mock trial exercises being an essential part of their preparation [17].

Additionally, many FOs have actively participated in DVI efforts during mass disasters. The results of the survey indicated that approximately half of the FOs surveyed (50.5%, 95% CI [43.7%, 57.3%]) had participated in DVI teams at the national level, while only 27.2%, 95% CI [21.5%, 33.5%] had participated in international DVI teams. A recent systematic review and meta-analysis emphasizes the critical role of FOs in DVI operations, demonstrating that dental identification can be used to identify at least 32% of victims in mass disaster scenarios [18]. This statistic underscores the importance of FOs in disaster response, where rapid and accurate identification is essential for providing closure to affected families and facilitating the legal and humanitarian aspects of disaster management.

### Professional support and networks

The recognition of FO as a distinct dental specialty differs across countries. In some regions, it is formally recognized as a specialty, while in others, it remains a sub-discipline within broader dental or forensic science.

It is concerning that only 40.3% (95% CI [33.8%, 47.1%]) of respondents indicated that FO is recognized as an independent specialty in their countries. This finding is reflected in the respondents' satisfaction levels, as only 16.5% expressed strong satisfaction regarding the support and recognition of FO in their respective countries. Nevertheless, the majority of participants expressed a favorable opinion of FO as a career choice, albeit with some reservations (59.2%, 95% CI [52.4%, 65.8%]).

Despite these challenges, multiple professional associations support the development of FO globally, with most FOs being members of such associations (75.6%, 95% CI [69.4%, 81.1%]). Among FOs who reported membership (*n* = 155), 38.7% (95% CI [31.3%, 46.5%]) were affiliated with the International Organization for Forensic Odonto-Stomatology (IOFOS), while 41.9% (95% CI [34.4%, 49.8%]) were part of the American Society of Forensic Odontology.

According to the IOFOS website (https://iofos.eu/member-of-iofos/), several FO associations and member organizations exist, as shown in Table 1. While the activity status of all these associations remains uncertain, their existence underscores a sustained interest in the field of FO. Generally, scientific associations play a crucial role by offering networking opportunities, fostering professional development, and providing platforms for advancing the discipline. National organizations play an important role in guiding dentists through approved pathways to gain experience in FO and practice within the legal frameworks of their respective countries. By adhering to recommended guidelines for good practice, these associations ensure that practitioners meet the standards expected in the field. Membership in these associations provides aspiring FOs with invaluable opportunities to learn from seasoned professionals, gaining practical insights into the profession.

### Foremost challenges in FO

An analysis of responses from FOs reveals the most pressing challenges hindering the advancement of the field A lack of recognition and awareness was identified as a significant concern by 21.8% (95% CI [16.6%, 27.9%]) of respondents. This issue is closely tied to limited career and job opportunities, highlighted by 18.4% (95% CI [13.6%, 24.2%]) of FOs. Other major challenges include education and training deficiencies (13.1%, 95% CI [9.0%, 18.2%]), resource and funding limitations (9.7%, 95% CI [6.2%, 14.3%]), and the pursuit of collaboration and standardization (10.7%, 95% CI [7.0%, 15.4%]). Specific technical challenges such as bite mark analysis (8.3%, 95% CI [5.1%, 12.6%]), dental age estimation (3.9%, 95% CI [1.9%, 7.2%]), and identification (2.9%, 95% CI [1.2%, 5.9%]) also emerged as key concerns.

Respondents expressed apprehension about the integration of technology and the future trajectory of FO, with 7.3% (95% CI [4.3%, 11.4%]) identifying these as significant issues. A small but noteworthy group (3.9%, 95% CI [1.9%, 7.2%]) did not provide any specific challenges.

The feedback gathered underscores several critical barriers that impede progress within FO, touching on issues such as recognition, career opportunities, education, funding, collaboration, and technical practice. A central theme emerging from the responses is the lack of both public and institutional recognition, which severely limits support, visibility, and overall credibility for the field. This lack of awareness, in turn, leads to a shortage of career opportunities, as many institutions and employers fail to fully recognize the specialized skills that FO professionals offer. Addressing these gaps by promoting greater recognition and creating clearer career pathways is essential. By doing so, the field can attract and retain skilled professionals, ensuring its continued growth, sustainability, and relevance in the global forensic landscape.

Education and training deficiencies emerged as significant obstacles in the field, with respondents highlighting gaps in the quality and accessibility of specialized programs, which aligns with findings from a recent study [5]. These deficiencies hinder practitioners’ ability to effectively manage complex cases and stifle the overall progress of the field. The challenges are further exacerbated by resource and funding limitations, which restrict not only research and professional development but also access to advanced technologies that could enhance the accuracy and innovation in FO.

Collaboration and standardization were identified as additional key priorities. A unified and standardized approach to methodologies would significantly enhance the reliability and consistency across practices, ensuring more accurate and dependable results. The absence of well-defined standards and a lack of coordinated collaboration could lead to variability in practices, potentially affecting case outcomes and hindering the broader acceptance of FO within the forensic community.

Specific technical challenges, particularly in bite mark analysis, dental age estimation, and identification, also demand significant attention. These areas require specialized skills, and in the absence of robust frameworks, their efficacy can vary greatly. Such inconsistencies raise concerns about the accuracy and reliability of FO techniques, especially in legal, humanitarian, and investigative contexts. Furthermore, the integration of emerging technologies presents both opportunities and challenges. Respondents emphasized the need for clear strategies to effectively harness technological innovations. Artificial intelligence (AI), for example, holds the potential to revolutionize dental record analysis, bite mark analysis, and dental age estimation. However, without careful and strategic integration, the full benefits of these advancements may remain untapped, limiting their impact on the field.

Expanding research into these critical areas could significantly broaden the scientific foundation of FO, enhancing its practical applications in law enforcement and disaster response efforts. The opinions expressed by academic members teaching FO, as highlighted in a recent study [19], emphasize the necessity of adopting a balanced approach—one that seamlessly integrates technological advancements with rigorous training and hands-on practical application.

### Limitations in survey dissemination

In interpreting the results of this pilot survey, it is important to acknowledge the potential limitations associated with the validation and sampling method employed. Validation is a step in survey-based research to assure the survey’s accuracy to answer the research question. Even so, this pilot study provides an initial exploratory overview of forensic odontologists' perspectives and lays the groundwork for future, more comprehensive research efforts.

The response rate obtained from the survey, although valuable for understanding the perspectives of participants reached through FO professional associations, closed WhatsApp® groups, professional LinkedIn® network and ResearchGate® network may not fully represent the diversity and distribution of FO on a global scale. The reliance on these specific channels for participant recruitment could have introduced selection bias, as individuals who are not affiliated with such groups or associations may not have been reached by our survey. Therefore, caution should be exercised in generalising the findings to the broader population of FOs worldwide.

During the process of survey dissemination, several limitations were encountered that influenced the outreach strategy. Despite our attempt to engage certain professional associations in disseminating our online survey, we encountered limitations in utilising their mailing lists for distribution. In response to our request for assistance, they clarified that their mailing lists are reserved exclusively for messages of organisational importance. Consequently, direct distribution of our survey through their channels was not feasible.

In the process of reaching out to potential respondents for our online survey, we encountered significant communication challenges. Despite our best efforts to connect with individuals via the INPAFO email list (https://www.inpafo.in/our-members), we faced a notable obstacle: many of our messages were returned undelivered, indicating that the intended recipients could not be reached. Numerous websites of Associations and scientific societies related to FO are no longer accessible, and some of these organisations may no longer be operational. (Table 1). In 2007, the Japanese Society of Forensic Dental Science (JSFDS) was established with over 750 members [20]. Despite this, our survey reached only two FOs in Japan. Similarly, although France has 85 judicial experts in FO listed on the official experts' site (www.cours.appel.justice.fr), we received only four responses from colleagues in that country.

It's important to acknowledge that our dissemination strategy for the online survey did not utilise any social media platforms, including Meta® (formerly Facebook®), X® (formerly Twitter®), or Instagram®. While social media platforms are commonly employed to reach diverse audiences and facilitate widespread participation in online surveys, our decision to forgo their use may have constrained the reach and diversity of our sample. By not tapping into the potential of social media channels, we may have missed opportunities to engage with specific demographic groups or scientific communities that are active on these platforms. As a result, the generalisability and representativeness of our findings may be limited. In addition to the limitations associated with the sampling method, it is important to note that not all FOs may be accessible through online professional networks such as LinkedIn® and ResearchGate®. The reliance on online platforms for participant recruitment may have excluded individuals who are not actively engaged or registered on these platforms.

The distribution method used via WhatsApp® may have constrained the survey's visibility to a narrow audience within the initiator's immediate network, potentially limiting exposure to a broader demographic of FOs. Additionally, the specialised nature of the survey topic focusing on demographics within FO could have deterred participation from individuals with limited interest or expertise in this specific area. Time constraints related to professional commitments, ongoing research projects, or personal obligations may have further restricted participant engagement during the survey period.

Moreover, certain recipients might have been unaware of the survey or perceived the topic as unrelated to their specific interests within FO. Survey fatigue, resulting from frequent survey requests in the field, may have also diminished willingness to participate.

Furthermore, the informal communication style inherent to WhatsApp® and concerns regarding privacy and confidentiality associated with personal messaging platforms likely contributed to respondent hesitation or non-participation. Finally, the considerable volume of messages and potential distractions on WhatsApp® could have caused respondents to overlook or postpone responding to the survey amidst competing activities on the platform. These challenges underscore the importance of refining survey design and distribution strategies to optimise participant engagement and response rates in future research endeavours focusing on demographics within FO.

### Recommendations for future development

Addressing these multifaceted challenges holistically could significantly strengthen FOs role and impact within forensic science and the broader justice system. Increasing the inclusion of FO in undergraduate dental programs could provide a more solid foundation for future practitioners. Additionally, enhancing support and recognition within the broader scientific community is crucial for the field’s growth and development. Standardizing educational pathways and fostering international collaboration will ensure FO professionals are better equipped to meet the growing demands of this vital field. To maximise the global collection of FO demographics, future research should forge partnerships with international FO associations, utilise diverse communication channels and social media platforms to enhance outreach, incentivise participation with access to findings or workshops, implement multilingual and user-friendly survey designs to ensure inclusivity, and adopt longitudinal data collection strategies to track demographic trends over time.

## Conclusion

Forensic Odontology, as a niche sub-specialty within dentistry, faces numerous challenges, including the lack of recognition as a specialty, limited educational opportunities, insufficient career support, and technical obstacles such as inadequate research and professional development resources for FO practitioners. Addressing these challenges is essential to ensure the continued growth and impact of the field.

Future research should prioritize expanding outreach efforts on a larger scale by utilizing diverse communication channels, including social media platforms, and adopting a multilingual approach to reach a broader and more representative global sample. This would help overcome current barriers in visibility and accessibility and ensure that the perspectives and needs of FO practitioners across various regions are adequately represented. By tackling these key issues, FO can advance toward a future where its contributions to forensic science are widely recognized, and its practitioners are better supported and equipped to meet the growing demands of this vital field. This holistic approach will strengthen the foundation of FO as a recognized and respected discipline, ultimately benefiting the fields of law enforcement, disaster victim identification, and justice.

## Key points

1. Comprehensive global data on forensic odontologists remain essential.
2. Significant disparities exist in the standardization of forensic odontology training worldwide.
3. Both technical and non-technical challenges impacting professional practice have been identified.
4. A unified global framework for professional recognition and standardized training curricula is imperative.

## Data Availability

The data supporting this study's findings are available on request from the corresponding author.

## Declarations

The study was carried out following the ethical standards of the Declaration of Helsinki (Finland).

## References

[1] INTERPOL. Disaster Victim Identification Guide. INTERPOL; 2023. Available: https://www.interpol.int/content/download/589/file/18Y1344%20E%20DVI_Guide.pdf [Accessed 26 January 2024].

[2] Yasar ZF, Durukan E, Buken E. The knowledge level of dentists in turkey about their potential role on the disaster victims identification (DVI) team. Disaster Med Public Health Prep. 2019;13(3):533–8. 10.1017/dmp.2018.111. 30417805 10.1017/dmp.2018.111

[3] Smitha T, Sheethal HS, Hema KN, Franklin R. Forensic odontology as a humanitarian tool. J Oral Maxillofac Pathol. 2019;23:409–13. 10.4103/jomfp.JOMFP_249_18. 10.4103/jomfp.JOMFP_249_18 PMC6503812 31110447

[4] Nuzzolese E. Missing people, migrants, identification, and human rights. J Forensic Odontostomatol. 2012;30(Suppl 1):47–59. 23221266

[5] Al Ghazi R, Gardner A, Mossey P, Abualhija D, Mc Gregor SS, Mânica S. A scoping review of websites for forensic odontology training programs. J Forensic Odontostomatol. 2024;42(2):87–102. 10.5281/zenodo.13474319. 39244769 10.5281/zenodo.13474319 PMC11446573

[6] Amoëdo O. L’art dentaire en médecine légale. Paris: Masson et Cie; 1898.

[7] Keiser-Nielsen S. Person identification by means of teeth. Am J Forensic Sci. 1981;2:189.

[8] Mânica S, Mânica G, Pandey H, Rodrigues LG, Marques Santiago B, Ferreira Silva R. The role of women in forensic odontology. Rev Bras Odontol Legal. 2022;9(1):2–24. 10.21117/rbol-v9n12022-425.

[9] Lorkiewicz-Muszyńska D, Przystańska A, Łabęcka M, Kruszelnicki A. Current status of forensic odontology education – the underestimation of needs? Dent Med Probl. 2013;50(2):217–22.

[10] Giannakopoulos K, Lambrou P, Kaklamanos EG, Aristotelous A. The anthropological process of identifying missing persons as a teaching method for increasing awareness in legal and forensic dentistry in the Republic of Cyprus. Forensic Sci. 2024;4(4):598–603. 10.3390/forensicsci4040041.

[11] Hag Ali S, Franco A, Nuzzolese E, Mânica S. Teaching of Forensic Dentistry in Khartoum. Sudan Oral. 2024;4(1):90–100. 10.3390/oral4010008.

[12] Shoro S, Syed FMS, Mânica S. Awareness and importance of forensic odontology amongst faculty members and students of dental institutes in Pakistan. Forensic Sci Int. 2020;2:100116. 10.1016/j.fsint.2020.100116.

[13] Baig MZ, Siddiqi KM, Jabeen N, Israr M, Ehsan MT, Rahman F. Awareness and compliance about forensic dentistry among dental professionals of twin cities of Rawalpindi-Islamabad: a questionnaire-based study. Pak Oral Dent J. 2014;34(2):4. Available from: http://podj.com.pk/archive/Jun_2014/PODJ-17.pdf.

[14] Seraj P, Hamrah MH, Homayoun F, Maisam A, Ghafary ES, Hosseini S, Khosrozadeh M. Awareness and Attitude of Forensic Odontology among Undergraduate Dental Students in Kabul University of Medical Sciences, Afghanistan. Indian J Forensic Med Toxicol. 2021;15(4):2127–34.

[15] Alegre Valente RP, Costa Ribeiro JM, Silva Sakamoto SP, Rodrigues R, Franco A, Paranhos LR. Survey on the dental age estimation practice among Brazilian experts. Rev Obs Econ Latinoam. 2024;22(2):1–15. 10.55905/oelv22n3-138.

[16] Silver W, Souviron R. Dental autopsy. Boca Raton, FL: CRC Press; 2009.

[17] Acharya AB. Teaching forensic odontology: an opinion on its content and format. Eur J Dent Educ. 2006;10(3):137–41. 10.1111/j.1600-0579.2006.00405.x. 16842587 10.1111/j.1600-0579.2006.00405.x

[18] Merdietio Boedi RM, Angelakopoulos N, Nuzzolese E, Pandey H, Mânica S, Franco A. Positive identification through comparative dental analysis in mass disaster: a systematic review and meta-analysis. Forensic Sci Med Pathol. 2024. 10.1007/s12024-024-00876-7. 39158821 10.1007/s12024-024-00876-7

[19] Mânica S, Gorza L. Forensic odontology in the 21st century - Identifying the opinions of those behind the teaching. J Forensic Leg Med. 2019;64:7–13. 10.1016/j.jflm.2019.03.006. 30878916 10.1016/j.jflm.2019.03.006

[20] Komuro T, Tsutsumi H, Izawa H, Katsumura S, Saitoh H, Sakurada K, Sato K, Furukawa A. Social contribution of forensic odontology in Japan. Jpn Dent Sci Rev. 2019;55(1):121–5. 10.1016/j.jdsr.2019.09.003. 31660092 10.1016/j.jdsr.2019.09.003 PMC6806645

